# The Effect of Ultrasonic Probes on the Ability to Inspect Adhesive Joints

**DOI:** 10.3390/ma18091946

**Published:** 2025-04-24

**Authors:** Jakub Kowalczyk

**Affiliations:** Faculty of Civil and Transport Engineering, Institute of Machines and Motor Vehicles, Poznan University of Technology, 60-965 Poznan, Poland; jakub.kowalczyk@put.poznan.pl; Tel.: +48-61-665-2248

**Keywords:** nondestructive testing, ultrasonic testing, adhesive, ultrasonic probe

## Abstract

Ultrasonic tests are widely used, both in laboratory and industrial settings, to assess the quality of joints, mainly welded joints. Studies are being carried out on the possibility of ultrasonic evaluation of adhesive joints. This study was conducted using signal analysis in the time and frequency domains. The ultrasonic probes used in the tests were selected on the basis of the properties of the test elements. For example, when testing welded joints, ultrasonic probes with a water delay line bounded by a thin diaphragm were used. Since adhesives have different acoustic properties, it is necessary to evaluate the capabilities of different ultrasonic probes to test adhesive joints. Tests were conducted for two different adhesives (cyanoacrylate and structural) and eight ultrasonic probes with a frequency range of 1.660 to 13.70 MHz. In the literature, no studies have analyzed the use of ultrasonic probes at such different frequencies. Frequency has the greatest effect on the attenuation of ultrasonic waves and the ultrasonic wavelength, and it was noted that the adhesive could cause a 25 percent change in the maximum frequency of the ultrasonic pulse. It was also found that it is necessary to make reference samples before ultrasonic testing of adhesive joints, since specific frequencies can produce erroneous signals for the selected adhesives.

## 1. Introduction

Adhesive joints are used in virtually all areas of modern industry [1], with a particularly large share used in the automotive industry. The use of bonding makes it possible to efficiently fabricate aluminum bodies [2]. A significant increase in the use of bonding in the construction of motor vehicles was noted as early as the 1960s [3], which made it possible to reduce the weight of vehicles and the cost of production and significantly accelerated the development of this joining technology.

Bonding is an interesting and widespread method of joining. As early as the 1960s, cyanoacrylate adhesives were used to join skin in securing wounds [4,5], and fibrin adhesives were used to join damaged kidneys [6]. Adhesive joints are widely used in aircraft construction, where they are an alternative to riveting for joining stringers to plating. Bonding is used to join roof elements in self-driving vehicles; to assemble windshields; to join body elements, side door panels, and engine covers; and to secure threads.

Using adhesives in modern industry has a number of advantages [7,8,9,10]. Adhesive joints, without destroying the materials to be joined, ensure uniform distribution of stresses and seal and protect against vibration, noise, corrosion, and water. In addition, adhesive joints allow for joining different materials (e.g., steel and aluminum) and for joining metals that are difficult to weld (e.g., aluminum sheets). The fact that bonding is often combined with sealing, which makes it possible to improve the appearance of vehicles (for example, buses), is also significant, as is the possibility of completing quick repairs under field conditions. Adhesive joints also have disadvantages and limitations, which include the need to prepare the surface before bonding (degreasing, activating) [11,12,13], the limited durability of adhesives before application, and the limited mechanical strength of the joints (generally up to 30 MPa). Another important limitation is the difficulty of assessing the quality of the shaped joint.

Currently used methods for evaluating the quality of adhesive joints are based mainly on strength. These are standardized methods commonly used for testing new adhesives and surface preparation methods. These methods are used to verify the results of numerical calculations, such as the extended finite element method (XFEM) or meshless methods [14]. Strength studies of adhesive joints were presented in one study [15], where a significant evolution in adhesive joints was noted, especially in the use of structural adhesives. Great emphasis was placed on learning about the fracture mechanics of adhesive joints. The evaluation of bonded aerospace (aircraft) structures was carried out by routine tensile (pr EN 2243-1) and peel tests (BS EN 2243-2 for metal-to-metal joints and BS EN 2243-3 for metal-to-metal honeycomb joints) to determine strength [16].

**Figure 1:**
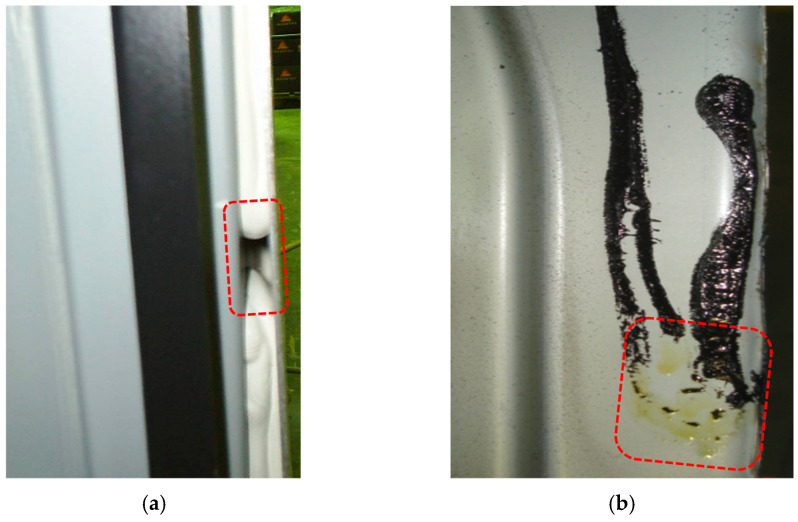
Defects in adhesives joints (red dotted boxes): (**a**) lack of adhesive in the joint; (**b**) significantly contaminated surface for bonding.

Gluing technology is well understood, but defects caused by various factors reduce the quality of adhesive joints. One such factor is the structural factor, which is relatively easy to reduce with modern calculation methods. The second cause of defects is errors by the workers applying the adhesive, for example, uncleaned surfaces or the absence of adhesive in places indicated by the designers (Figure 1).

Taking into account the contribution of adhesive joints in the construction of aircraft, vehicles, and other objects, it is extremely important to improve nondestructive methods for inspecting such joints.

**Figure 2:**
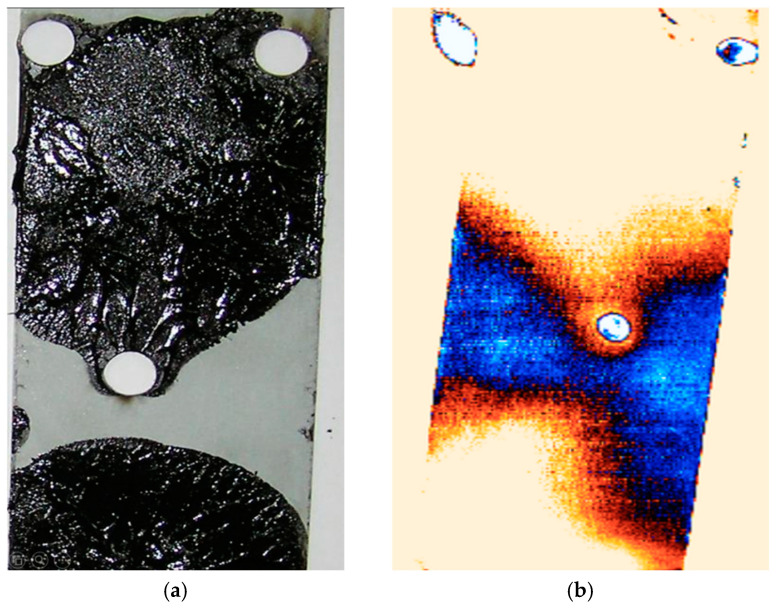
Adhesive joint tested by thermographic method: (**a**) view of joint after tearing; (**b**) image from thermographic camera.

Nondestructive methods of condition assessment are of particular importance in technology, since they enable inspection without interference with the materials under test, which means that they can be used not only during production but also during operation. The main nondestructive methods are visual (VT) [17], magnetic powder (MT) [18], ultrasonic (UT) [19], penetrant testing (PT) [20], X-ray (RT) [21], and thermographic (IR) methods [22,23]. To test adhesive joints, ultrasound and thermographic methods are mainly used. To a limited extent, the VT method can be used, but only for testing adhesive joints where the adhesive efflux can be observed and when joining transparent parts. Examples of adhesive joints with right-angled efflorescence indicating the presence of adhesive are shown in Figure 2. Notably, in the section marked with a dotted line, no adhesive was found despite the correct efflorescence. However, the application of this method is very limited.

The thermographic method uses the phenomena of temperature gradient, allowing for a quick assessment of adhesive joints, but this is mainly for parts of a small size. In the thermographic method, the condition of the surface to be tested is of great importance. An erroneous result can be obtained even by changing surface colors or the lighting in the tested room. An example of the result of a thermographic test of an adhesive joint is shown in Figure 2.

The ultrasonic method is widely used in industry as a nondestructive testing method based on the phenomena of reflection, transition, and refraction of ultrasonic waves. On the one hand, defects (cracks, shrinkage cavities, etc.) are detected, while on the other hand, this method allows the evaluation of material properties, measurement of residual stresses, and assessment of adhesive joints. Previous studies have confirmed the possibility of testing the quality of adhesive joints using ultrasonic methods. Different types of ultrasonic waves have been used to test adhesive joints; for example, the authors of [24] used low-frequency Lamb waves in pitch–catch mode or high-frequency longitudinal waves in pulse–echo mode. They not only showed the possibilities of using waves to test adhesive joints but also identified their advantages and disadvantages. However, in this work, analysis and testing were not carried out using different ultrasonic wave frequencies. This is particularly important because the frequency of an ultrasonic wave has the greatest impact on the attenuation of the wave and the wavelength. Wave attenuation determines the possibility of obtaining good signals, while wavelength affects the resolution of the measurement system (measurement error). Similarly, the work of [25] was limited to a narrow range of frequencies, while using other measures of ultrasound. The authors found that exploration of the first interface reflection is least likely to correctly determine the size of the defect. For sizing, the second and third interface reflections show better performance for inclusions and delaminations, respectively. The fourth reflection is characterized by signal attenuation and a decrease in ultrasonic feature performance. What is important, however, is that the possibility of obtaining the third and fourth echoes largely depends on the frequency of the wave. The authors of [26] also conducted an interesting study on adhesive joints using the ultrasonic method. A narrow frequency range was used, but the authors also used an X-ray technique as a second method. The combination of these two methods made it possible to assess the quality of adhesive joints in aluminum samples. The use of the X-ray method in steel specimens has been very limited. The authors of [27] also utilized aluminum samples and probes with a very limited range of frequencies. They used an ultrasonic head with a manufacturer-declared frequency of 5 MHz. The authors of [28] used ultrasonic waves to support bonding technology. Waves of a significantly higher power than those used in nondestructive testing were used. This is important because, when planning tests of adhesive joints that have not fully set, it is necessary to select probes and test conditions so that ultrasonic waves do not reduce the quality of the joints [29].

While the possibility of ultrasonic testing of adhesive joints is confirmed, the conditions and rules for conducting such tests are not widely known. The purpose of the present work is to evaluate the possibility of using longitudinal ultrasonic waves of different frequencies to test adhesive joints for different groups of adhesives.

## 2. Research

### 2.1. Research Procedure

**Figure 3:**
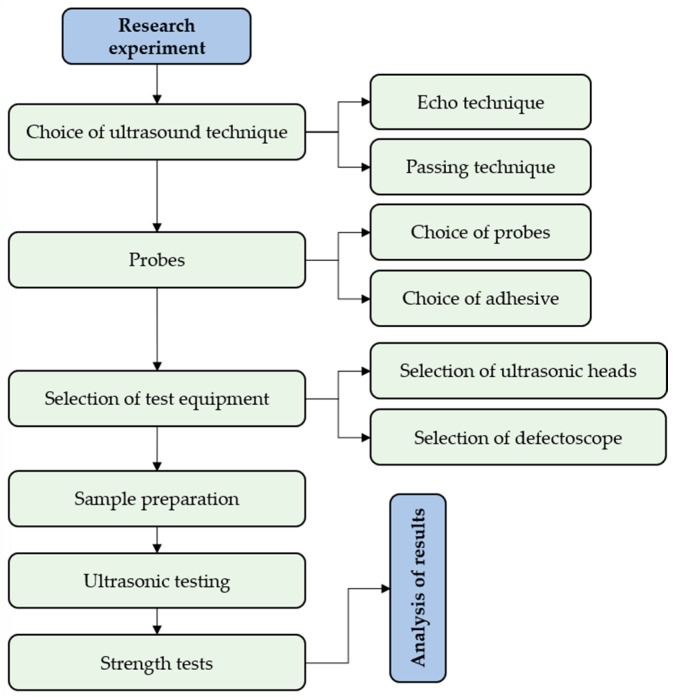
Research plan.

The entire study was carried out according to the plan shown in Figure 3. First, the ultrasonic technique was selected; then, the type of samples (bonding material, shape, and adhesive) was determined. After selecting the type of samples, the ultrasonic apparatus (defectoscope and ultrasonic probes) was selected. In the following steps, the samples were prepared, and they were subjected to ultrasonic testing and strength testing. Finally, the results were analyzed.

### 2.2. Materials

Steel specimens were used in the study, since steel has the largest share in the construction of vehicles and machinery [30,31,32]. It was decided to use carbon steel 1.0503. The chemical composition of steel 1.0503 is shown in Table 1. Various adhesives were considered to join the steel discs, but two were decided upon: a cyanoacrylate adhesive and a high-strength, two-component structural adhesive. Both adhesives are industrial adhesives designed for joining steel, and the bond should be as thin as possible.

**Figure 4:**
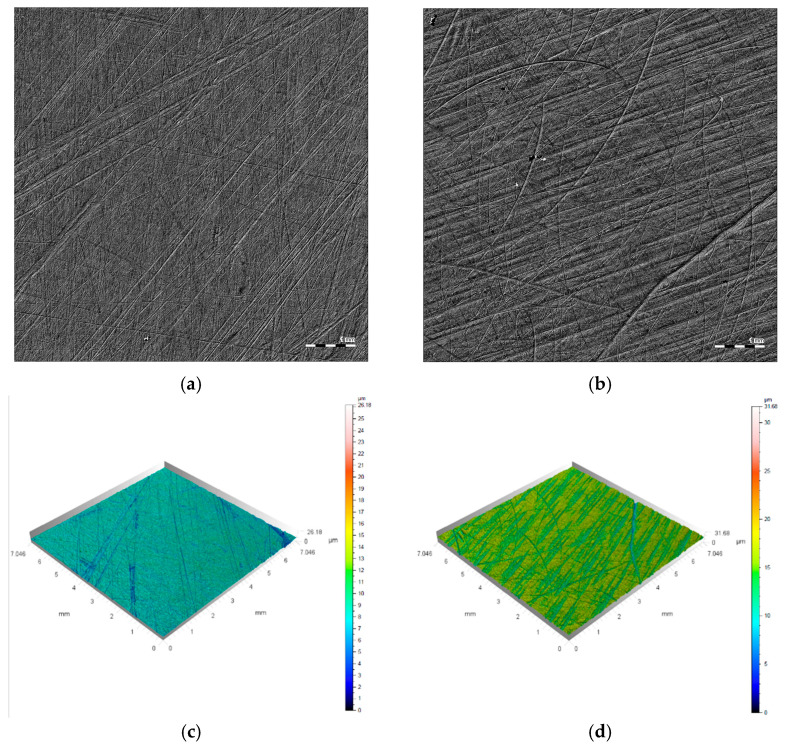
Example of tested samples’ surfaces: (**a**,**b**) magnification view; (**c**,**d**) roughness.

The surface to be glued was sanded with sandpaper and then degreased with a degreaser recommended by the adhesive manufacturer. An example view of the surface is shown in Figure 4a,b, and the roughness of these surfaces is shown in Figure 4c,d.

**Figure 5:**
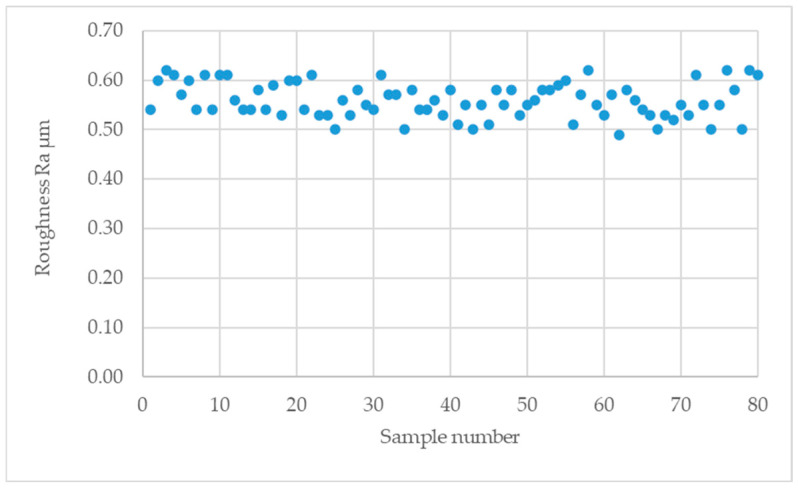
The value of the surface roughness parameter of the tested samples, Ra.

The results of roughness measurements for all samples are shown in Table A1 of the Appendix A, while a graph form is shown in Figure 5.

The average Ra value was 0.56 µm, the lowest recorded value was 0.49 µm, and the highest was 0.62 µm. Due to the small differences in roughness, the condition of the surface does not affect the height of the pulses obtained on the screen of the ultrasonic defectoscope [33].

**Figure 6:**
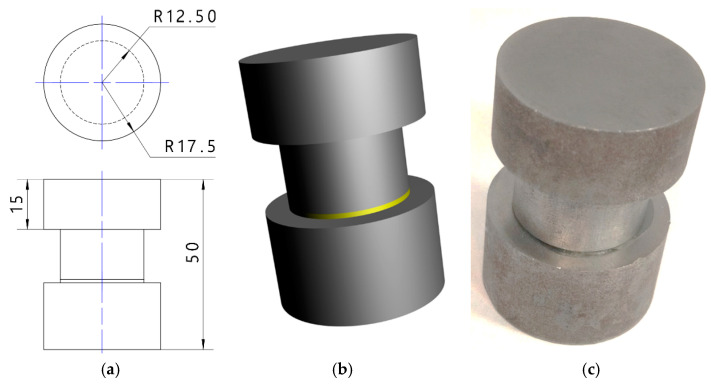
Sample used in correlation studies: (**a**) sample drawing; (**b**) sample model; (**c**) sample view.

The specimens shown in Figure 6 were used for testing, and the proposed shape enabled not only useful signals for ultrasonic testing but also efficient measurement of the tensile force during strength testing. When gluing the steel specimens, a template was used to ensure axial alignment of the specimens. For some of the specimens, the glued surface was not degreased in order to reduce their quality (tensile strength).

### 2.3. Research Methods

The ultrasonic echo technique was used in this study, which has practical industrial applications. The research was carried out on a classic digital defectoscope CUD, which allows for reading the actual frequency of the ultrasonic probe and retrieving the obtained signals. Downloading the signals to a computer allows for more extensive evaluation.

Ultrasonic probes with different frequencies that can take measurements for steel were selected for the test. They were selected for the frequency range from 1.66 to 13.70 MHz. Ultrasonic testing using probes in the 2–20 MHz frequency range has been conducted in a variety of areas, including to evaluate coating–substrate adhesion joints [34,35] and adhesive joints, as well as spot-welded joints [36,37]. A list of the probes and their properties is shown in Table 2, and the results of ultrasonic probe frequency measurements are shown in Table A2 in the Appendix A. Attempts were made with other probes, such as those at 500 kHz and 1 MHz (specified by the probe manufacturers), but the measurement system was characterized by a low resolution. Heads with higher frequencies (20 MHz) for the materials tested were characterized by too much attenuation.

**Figure 7:**
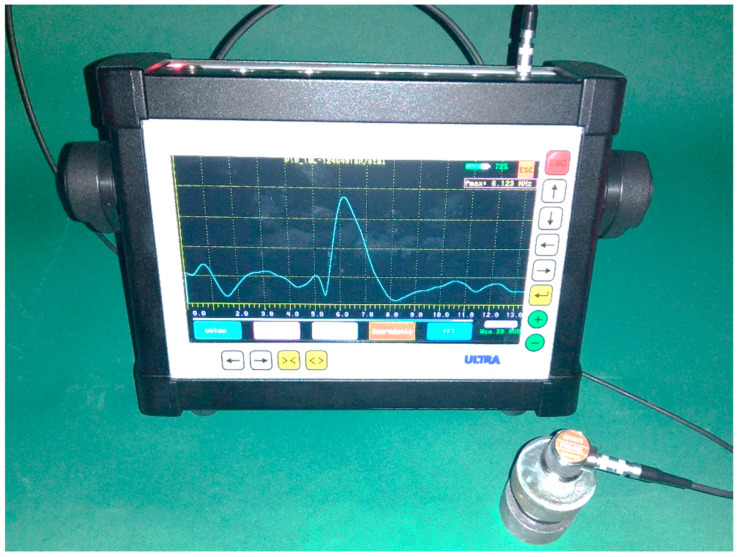
Test stand: determination of the actual frequency of the ultrasonic probe.

The view of the measuring system is shown in Figure 7. One of the advantages of using the defectoscope is the possibility of recording the obtained signal and its further analysis on external systems.

**Figure 8:**
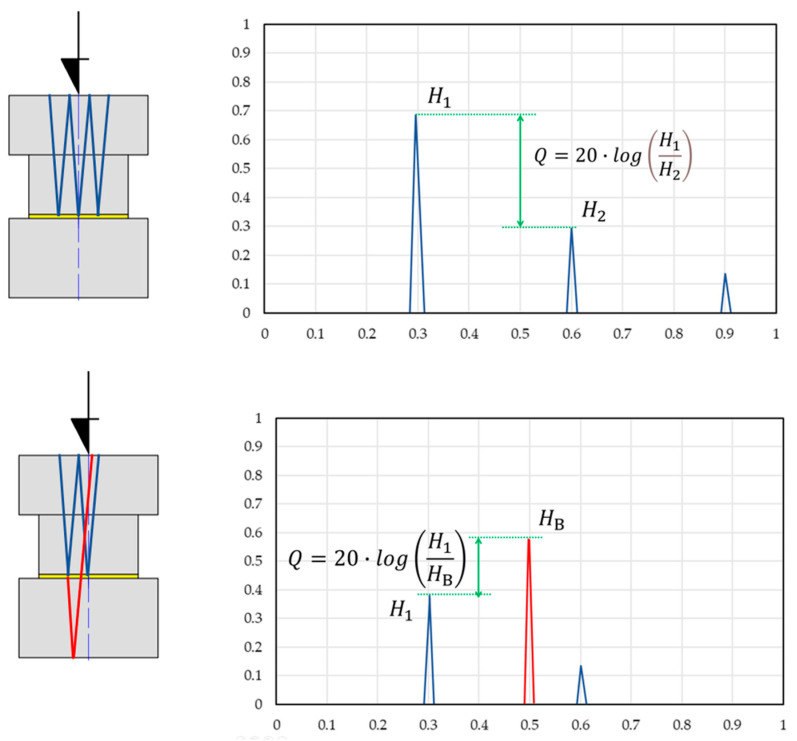
Measurement principle for adhesives with different attenuation. Q—ultrasonic measure, H_1_—height of the first pulse from the joint area, H_2_—height of the second pulse in the joint area, HB—height of the pulse from the bottom of the sample.

Two different types of adhesives were used in the tests, resulting in two different types of signals on the screen of the ultrasonic defectoscope. In the first, a sequence of pulses was obtained from the joint area; in the second, a sequence of pulses from both the joint area and the bottom of the sample was obtained (Figure 8). Only the first two pulses obtained on the screen of the defectoscope were used for analysis.

The drop in the height of the first two pulses (in decibels) obtained on the screen of the defectoscope was used as an ultrasonic measure of the quality of the adhesive bond. For a high-strength structural adhesive, due to the lack of pulses coming from the bottom of the sample, the heights of pulses from the joint area were used to determine the ultrasonic measure. For the cyanoacrylate adhesive, the pulses from the joint area and the bottom of the sample were considered for the low-frequency ultrasonic wave. It is worth noting that the appearance of a pulse from the bottom of the sample already indicates a high-quality bond.

**Figure 9:**
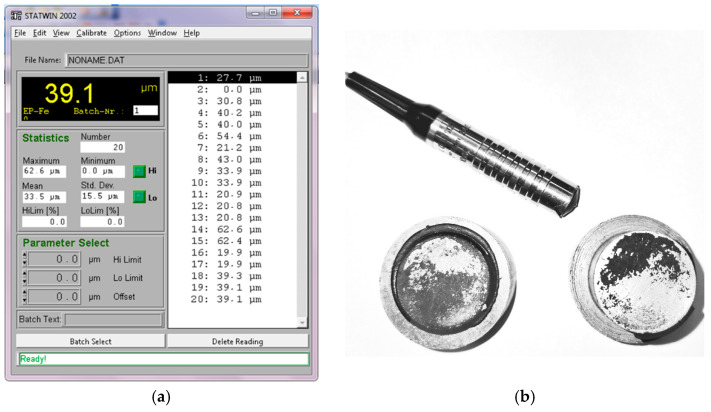
Adhesive thickness measurements: (**a**) view of STATWIN program window; (**b**) view of the leptoscope and glue sample.

As part of the study, the thickness of the adhesive layer was evaluated. For this purpose, a Karl Deutsch leptoscope, Karl Deutsch, Wuppertal, Germany was used together with the STATWIN 2002 program (Figure 9). The average thickness of the cyanoacrylate adhesive was 6.5 µm, while the structural adhesive was five times greater at 33.0 µm. A summary of the adhesive thickness measurement results is presented in Table A3 and Table A4 in Appendix A.

## 3. Results and Discussion

After adhesive bonding, ultrasonic tests were performed for all samples. Measurements were recorded for all ultrasonic probes used. According to the adopted test methodology, ultrasonic measures in both the time and frequency domains were recorded.

Measurements in the time domain were carried out first. Table 3 presents a summary of the results for the structural adhesive for all the ultrasonic probes used, while Table 4 shows these results for the cyanoacrylate adhesive.

During the measurements, the maximum frequency was also recorded. For this purpose, the Fast Fourier Transform was used. The results for the cyanoacrylate adhesive are presented in Table 5, and those for the structural adhesive are shown in Table 6.

The results of the strength tests are shown in Table 7. During the tests, 20 specimens of cyanoacrylate adhesive and 20 specimens of structural adhesive were torn. In each group, only half of the samples had their surfaces degreased. During the strength tests, the maximum breaking force of the joint was recorded, and the tensile stresses were determined based on the surfaces properties of the steel discs. The results are shown in Table 7.

**Figure 10:**
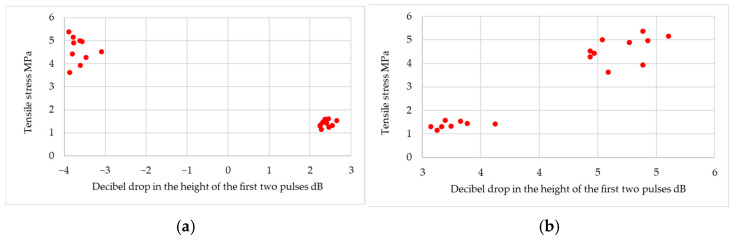
Example diagrams for cyanoacrylate adhesive: (**a**) for ultrasonic probe DS12HB0.8-3; (**b**) for ultrasonic probe DS6HB4-12.

**Figure 11:**
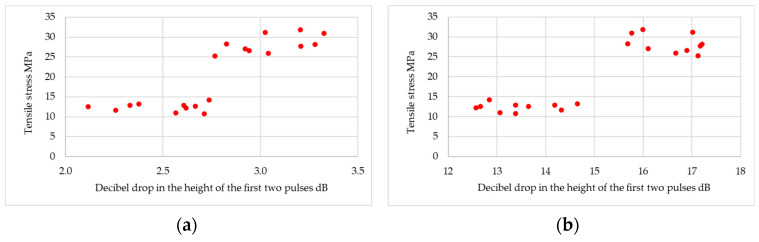
Example diagrams for structural adhesive: (**a**) for ultrasonic probe DS12HB0.8-3; (**b**) for ultrasonic probe P10-10L.

The experiment was able to obtain useful results. First, correlations between ultrasonic measures and the quality of adhesive joints were determined. In the study of the cyanoacrylate adhesive, it was found that, for frequencies up to 7.5 MHz, a pulse was obtained that originated from the bottom of the tested samples. It was observed that, for frequencies below 7.5 MHz, as the ultrasonic measure increases, the quality of the joint decreases; example results in the form of a graph are shown in Figure 10 and Figure 11. It was observed that the attenuation of the ultrasonic wave at the lowest frequency is so small that the pulse from the bottom of the sample is higher than the pulse from the joint areas. Such an arrangement of pulses generates an ultrasonic measure below zero.

Example results for the structural adhesive are shown in Figure 11.

**Figure 12:**
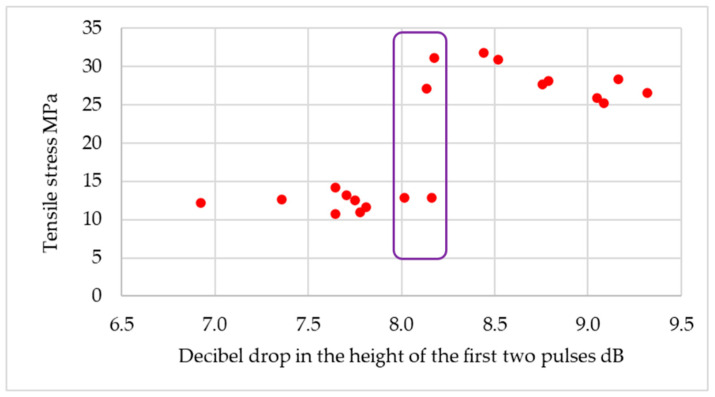
Results obtained for measurements using MB2S probe for structural adhesive.

For some of the ultrasonic probes used, it was not possible to confidently assess the quality of the joint based on ultrasonic measurements. For example, for the MB2S probe, for a drop in decibels of about 8 dB for two pulses, the tensile stresses ranged from about 12 to 30 MPa (Figure 12).

**Figure 13:**
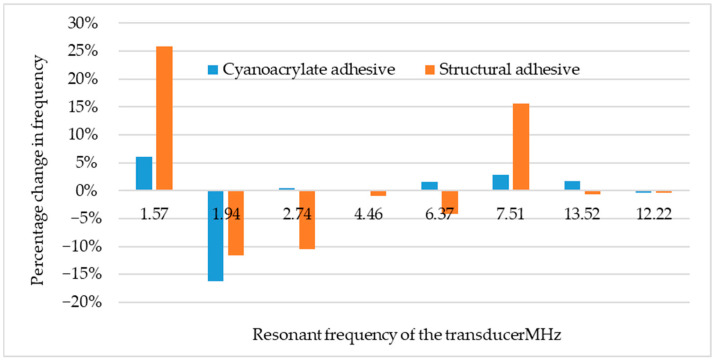
Frequency variation of the ultrasonic wave, depending on the type of glue and the output frequency.

No effect of the quality of the joint on the maximum frequency of the ultrasonic wave was noted. However, an effect of the presence of an adhesive was observed on the frequency of the ultrasonic wave pulse from the adhesive joint area. The largest difference was more than 25 percent for a probe with a transducer frequency of 1.57 MHz. No significant differences in frequencies were noted for probes with frequencies above 10 MHz or for a probe with a frequency of 4 MHz. It can be assumed that the glue in the sample acts as a filter, dampening certain frequencies. All results are shown in Figure 13.

The measurements were repeated several times. Confidence intervals were determined with a significance level of 95%. In my opinion, the adhesives act as a filter. This filter causes attenuation of certain frequency components of the ultrasonic wave. In addition, there is a relationship between the wavelength and frequency and the thickness of the glue joints. Wavelength is proportional to wave speed and inversely proportional to wave frequency, while the wave speed depends on the material. For the adhesives used in this study, the speed of the ultrasonic wave is virtually unmeasurable. In accordance with the technology recommended by the manufacturers of adhesives, adhesive joints of different thicknesses were prepared (one was for cyanoacrylate adhesives, and another for structural adhesives). Hence, the differences are clear, but at the same time, no trend was observed.

When planning a study, the following must be considered. Materials have the greatest influence on the pulse height and even have an effect on obtaining the desired signals. Steel is a material that weakly attenuates ultrasonic waves. Non-ferrous metals (e.g., aluminum) attenuate waves much more strongly, but testing is possible. Plastics attenuate waves so strongly that the use of classical ultrasonic heads is virtually impossible.

## 4. Conclusions

The purpose of the study was to evaluate the feasibility of using longitudinal ultrasonic waves at different frequencies to test adhesive joints for different groups of adhesives.

Tests were conducted for two different adhesives—cyanoacrylate adhesives and structural adhesives—and eight ultrasonic probes with a frequency range of 1.660 to 13.70 MHz were used. This is particularly important because researchers have so far been limited to a narrow frequency range, overlooking the aspect of how frequency affects research feasibility.

The results confirmed that it is possible to obtain useful signals using a defectoscope for the ultrasonic evaluation of adhesive joints. For the cyanoacrylate adhesive, at a low frequency, it was possible to obtain pulses of ultrasonic waves reflected from the bottom of the test sample.

The samples used in the tests had varying tensile strengths, ranging from 1.2 to 5 MPa for the cyanoacrylate adhesive and from 10.8 to 31.8 MPa for the structural adhesive.

For some ultrasonic probes, it was possible to obtain pulses of ultrasonic longitudinal waves not only from the joint area but also from the bottom of the test specimen. The glue used to make the joints acted as a filter, changing the frequency of the ultrasonic wave. For the structural adhesive, the frequency of the wave for the ultrasonic probe with a resonant frequency of 1.57 MHz increased by 25 percent. Cyanoacrylate glue affected the maximum frequency of the wave to a lesser extent.

The results confirmed that for each type of adhesive and the probes used, reference samples should be prepared, because it is not always possible to associate the quality of the joint with ultrasonic quality measures.

Cyanoacrylate glue is harder and has a much thinner, sometimes even negligible, bond, while structural adhesives are more flexible and are thicker. This causes them to attenuate high-frequency waves much more strongly. It is not possible to conduct such tests at low frequencies, because the resolution of the measurement system significantly decreases, and the length of the dead zone increases. Since cyanoacrylate glue has low attenuation, the wave and its associated energy can pass quite freely through the joint twice (once from the top to the bottom of the sample, and the second time returning to the head upwards). There is a possibility that some ultrasonic heads will pass the wave through the structural adhesive once, but only with the use of a transmission technique. Such a technique has no practical industrial application and was not an object of study in this work.

The results confirmed that there is high potential for the use of ultrasonic inspection in industrial settings, particularly to detect defects in adhesive joints in the automotive industry. Since modern car bodies are made mainly of steel, and less often of aluminum, it is possible to control the quality of the joints (in terms of the location of the adhesive, and in selected examples, even in terms of the quality of the joint).

## Figures and Tables

**Table 1:** Chemical composition of steel 1.0503 (%).

C	Si	Mn	Cr	Ni	Mo	Cu	S	P
0.42	0.10	0.50	max.	max.	max.	max.	max.	max.
0.50	0.40	0.80	0.30	0.30	0.10	0.30	0.04	0.04

**Table 2:** Parameters of the ultrasonic transducer used.

Parameter		DS12HB0.8-3	DS12 HB 1-6	MB2S	MB4S	P10-10L	S6WB10WM	SDS PB 6-16	DS6HB4-12
Custom labeling		DS1	DS2	MB2	MB4	P10	S6	SDS	DS3
Manufacturer		KD	KD	GE	GE	KD	KD	KD	KD
Frequency	MHz	1.57	1.66	2.75	4.45	6.37	7.68	12.21	13.70
Diameter of the transducer	mm	12	12	10	10	10	6	6	3
Effective diameter	mm	11.64	11.64	9.7	9.7	9.7	5.82	5.82	2.91
Wave speed of tested material	m/s	6001	6001	6001	6001	6001	6001	6001	6001
Wavelength	mm	3.83	3.61	2.19	1.35	0.94	0.78	0.49	0.44
Near field	mm	7.9	8.5	10.2	17.1	24.7	10.6	17.1	4.7
Coefficient of decrease in decibels, K	-	0.87	0.87	0.87	0.87	0.87	0.87	0.87	0.87
Sine of the angle of the divergence of the beam	-	0.3	0.3	0.2	0.1	0.1	0.1	0.1	0.1
Divergence angle	°	16.6	15.6	11.3	6.9	4.8	6.7	4.2	7.5
Distance from the transducer	mm	30	30	30	30	30	30	30	30
Beam width	mm	17.93	16.79	12.00	7.31	5.09	7.06	4.42	7.93

**Table 3:** Decibel dB pulse height drop for the structural adhesive.

	Decibel (dB) Pulse Height Drop							
No.	DS1	DS2	MB2	MB4	P10	S6	SDS	DS3
1	2.9	4.1	8.1	13.2	16.1	18.9	21.5	19.7
2	2.8	3.9	9.2	12.1	15.7	19.1	19.6	23.3
3	3.3	3.9	8.8	12.7	17.2	18.2	20.2	24.3
4	2.8	3.5	9.1	12.7	17.1	16.7	23.3	24.8
5	3.2	3.7	8.8	12.9	17.2	19.4	25.1	21.8
6	2.9	3.8	9.3	13.1	16.9	16.5	20.8	25.0
7	3.3	3.8	8.5	13.1	15.8	17.6	20.5	23.8
8	3.2	4.0	8.4	13.6	16.0	16.9	22.4	25.5
9	3.0	3.6	9.0	12.9	16.7	17.7	19.8	22.8
10	3.0	3.7	8.2	12.7	17.0	17.4	20.8	21.4
11	2.1	2.9	7.8	11.0	13.6	16.0	17.5	17.2
12	2.6	3.3	6.9	10.8	12.6	15.9	18.9	18.7
13	2.6	3.5	8.0	11.2	13.4	14.9	17.7	16.4
14	2.7	3.1	7.6	11.4	12.8	14.0	18.6	19.9
15	2.7	2.9	7.4	10.8	12.7	13.6	17.3	18.4
16	2.6	3.2	7.8	10.8	13.1	15.1	19.1	18.6
17	2.3	3.2	7.8	10.8	14.3	13.4	18.3	19.6
18	2.4	3.3	7.7	11.4	14.6	14.6	16.8	19.7
19	2.7	3.0	7.6	11.3	13.4	14.3	15.7	17.2
20	2.3	3.2	8.2	11.7	14.2	14.8	18.9	19.3

**Table 4:** Decibel dB pulse height drop for cyanoacrylate adhesive.

	Decibel (dB) Pulse Height Drop							
No.	DS1	DS2	MB2	MB4	P10	S6	SDS	DS3
1	−3.5	1.1	5.6	4.9	13.5	1.7	3.6	4.4
2	−3.8	1.0	4.2	4.6	14.2	1.9	4.1	4.8
3	−3.9	1.4	3.4	5.0	13.5	1.8	4.1	4.9
4	−3.9	1.3	5.6	4.6	13.1	1.6	3.9	4.6
5	−3.1	1.3	6.7	4.9	13.7	1.6	3.8	4.4
6	−3.6	1.3	7.4	4.8	14.4	1.9	3.8	4.5
7	−3.6	1.4	4.8	4.4	14.1	1.9	3.6	4.9
8	−3.6	1.4	5.9	4.7	13.6	1.8	4.1	4.9
9	−3.8	1.5	2.8	4.9	13.7	1.7	4.1	4.5
10	−3.8	1.4	5.9	4.4	13.9	1.7	4.0	5.1
11	2.2	5.6	8.4	10.5	19.0	8.8	2.2	3.1
12	2.5	5.5	8.6	11.1	20.8	8.6	2.3	3.2
13	2.5	5.8	8.7	11.0	20.5	8.6	2.4	2.9
14	2.2	5.8	8.3	10.6	20.7	8.7	2.0	3.2
15	2.5	6.0	8.5	10.6	19.4	8.9	2.4	3.0
16	2.7	5.9	8.7	11.0	20.5	8.9	2.1	3.3
17	2.3	5.6	8.3	10.7	20.2	8.9	2.3	3.1
18	2.3	5.8	8.3	10.7	20.6	8.7	2.0	3.4
19	2.4	5.5	8.6	10.9	20.5	8.8	2.3	3.6
20	2.4	5.7	8.4	10.9	20.4	8.6	2.4	3.2

**Table 5:** Maximum frequency of the ultrasonic wave pulse when passing through cyanoacrylic adhesive.

	Maximum Frequency of the Ultrasonic Wave Pulse When Passing Through Cyanoacrylic Adhesive, MHz							
No.	DS1	DS2	MB2	MB4	P10	S6	SDS	DS3
1	1.65	1.73	2.69	4.48	6.82	7.35	14.05	12.11
2	1.65	1.68	2.78	4.48	6.76	7.49	13.30	12.28
3	1.64	1.75	2.75	4.38	6.69	7.81	13.60	12.12
4	1.69	1.74	2.76	4.53	6.37	7.67	13.97	12.21
5	1.66	1.78	2.67	4.45	6.21	8.00	13.58	12.25
6	1.65	1.45	2.82	4.52	6.25	8.02	13.29	12.25
7	1.68	1.76	2.75	4.51	6.14	7.68	13.94	12.13
8	1.64	1.67	2.80	4.46	6.70	7.83	13.93	12.25
9	1.67	1.57	2.68	4.47	6.28	8.04	13.82	12.20
10	1.66	1.75	2.80	4.45	6.54	7.35	13.34	12.13
11	1.66	1.51	2.79	4.40	6.63	7.70	13.55	12.19
12	1.69	1.58	2.75	4.48	6.07	8.04	14.04	12.15
13	1.69	1.45	2.69	4.50	6.33	7.76	13.48	12.12
14	1.68	1.39	2.83	4.37	6.53	7.37	13.98	12.21
15	1.65	1.71	2.82	4.43	6.75	7.64	13.91	12.09
16	1.69	1.76	2.79	4.47	6.29	8.14	13.94	12.24
17	1.64	1.65	2.66	4.48	6.05	8.01	13.55	12.15
18	1.65	1.37	2.73	4.43	6.36	7.55	14.02	12.13
19	1.69	1.38	2.81	4.44	6.77	7.62	13.87	12.32
20	1.68	1.80	2.72	4.43	6.79	7.25	14.00	12.09

**Table 6:** Maximum frequency of the ultrasonic wave pulse when passing through the structural adhesive.

	Maximum Frequency of the Ultrasonic Wave Pulse When Passing Through Structural Adhesive, MHz							
No.	DS1	DS2	MB2	MB4	P10	S6	SDS	DS3
1	1.97	1.74	2.44	4.42	6.12	8.73	13.49	12.06
2	1.97	1.91	2.48	4.42	6.12	8.59	13.45	12.19
3	1.97	1.69	2.50	4.43	6.12	8.78	13.46	12.04
4	1.99	1.53	2.45	4.45	6.11	8.64	13.42	12.13
5	1.99	1.67	2.47	4.40	6.02	8.71	13.42	12.07
6	1.96	1.86	2.47	4.44	6.05	8.76	13.41	12.16
7	1.99	1.85	2.42	4.40	6.09	8.68	13.42	12.26
8	1.97	1.64	2.43	4.40	6.06	8.65	13.41	12.24
9	1.99	1.51	2.41	4.45	6.09	8.56	13.42	12.14
10	1.97	1.67	2.49	4.40	6.22	8.66	13.41	12.11
11	1.99	1.64	2.49	4.41	6.06	8.71	13.50	12.27
12	1.99	1.71	2.45	4.44	6.11	8.77	13.43	12.22
13	1.99	1.78	2.41	4.40	6.09	8.68	13.42	12.23
14	1.96	1.77	2.48	4.44	6.10	8.76	13.46	12.18
15	1.97	1.50	2.45	4.39	6.07	8.71	13.41	12.28
16	1.96	1.62	2.45	4.39	6.11	8.58	13.40	12.31
17	1.98	1.66	2.50	4.41	6.10	8.65	13.42	12.15
18	1.98	1.79	2.45	4.45	6.15	8.56	13.44	12.14
19	1.98	1.87	2.45	4.41	6.17	8.60	13.46	12.01
20	1.96	1.85	2.45	4.42	6.17	8.73	13.49	12.24

**Table 7:** Strength test results.

Cycnacrylate Adhesive		Structural Adhesive	
No.	Tensile Stress, MPa	No.	Tensile Stress, MPa
1	4.3	1	27.1
2	4.9	2	28.3
3	5.4	3	28.1
4	3.6	4	25.3
5	4.5	5	27.7
6	5.0	6	26.6
7	3.9	7	30.9
8	5.0	8	31.8
9	4.4	9	25.9
10	5.2	10	31.2
11	1.3	11	12.5
12	1.3	12	12.2
13	1.3	13	12.9
14	1.3	14	14.2
15	1.6	15	12.6
16	1.5	16	11.0
17	1.2	17	11.6
18	1.4	18	13.2
19	1.4	19	10.8
20	1.6	20	12.9

## Data Availability

The data presented in this study are available on request from the corresponding author.

## Appendix A

**Table A1:** Summary of the roughness parameter, Ra, for all samples.

	Roughness Parameter, Ra (µm)	
No.	30 mm	20 mm
1	0.54	0.51
2	0.6	0.55
3	0.62	0.5
4	0.61	0.55
5	0.57	0.51
6	0.6	0.58
7	0.54	0.55
8	0.61	0.58
9	0.54	0.53
10	0.61	0.55
11	0.61	0.56
12	0.56	0.58
13	0.54	0.58
14	0.54	0.59
15	0.58	0.6
16	0.54	0.51
17	0.59	0.57
18	0.53	0.62
19	0.6	0.55
20	0.6	0.53
21	0.54	0.57
22	0.61	0.49
23	0.53	0.58
24	0.53	0.56
25	0.5	0.54
26	0.56	0.53
27	0.53	0.5
28	0.58	0.53
29	0.55	0.52
30	0.54	0.55
31	0.61	0.53
32	0.57	0.61
33	0.57	0.55
34	0.5	0.5
35	0.58	0.55
36	0.54	0.62
37	0.54	0.58
38	0.56	0.5
39	0.53	0.62
40	0.58	0.61

**Table A2:** Measurements of the average frequency of ultrasonic probes.

	Frequency (MHz)							
No.	DS1	DS2	MB2	MB4	P10	S6	SDS	DS3
1	1.94	1.67	2.74	4.46	6.37	7.51	13.52	12.22
2	1.53	1.66	2.76	4.45	6.37	7.56	13.85	12.20
3	1.29	1.67	2.75	4.46	6.38	7.49	13.93	12.21
4	1.46	1.66	2.75	4.46	6.37	7.84	13.43	12.21
5	1.23	1.65	2.74	4.44	6.38	7.79	14.09	12.23
6	1.39	1.66	2.73	4.45	6.38	7.89	14.10	12.23
7	1.38	1.67	2.76	4.45	6.37	7.77	13.57	12.20
8	1.65	1.66	2.77	4.46	6.37	7.59	13.35	12.23
9	1.45	1.67	2.73	4.46	6.38	7.48	13.47	12.20
10	1.41	1.67	2.73	4.45	6.37	7.70	13.61	12.22
11	1.65	1.66	2.76	4.45	6.37	7.55	13.68	12.22
12	1.58	1.67	2.75	4.45	6.37	7.61	13.22	12.23
13	1.37	1.66	2.73	4.45	6.37	7.68	13.48	12.22
14	1.60	1.67	2.73	4.46	6.38	7.82	13.66	12.20
15	1.89	1.66	2.76	4.44	6.37	7.53	13.46	12.23
16	1.44	1.67	2.76	4.45	6.37	7.82	13.70	12.21
17	1.45	1.67	2.75	4.45	6.37	7.60	14.11	12.22
18	1.28	1.66	2.75	4.46	6.37	7.65	13.19	12.22
19	1.87	1.66	2.74	4.45	6.38	7.77	14.00	12.21
20	1.29	1.67	2.75	4.44	6.37	7.78	13.65	12.22
21	1.48	1.65	2.76	4.46	6.37	7.82	14.03	12.20
22	1.81	1.67	2.76	4.45	6.37	7.88	14.01	12.20
23	1.85	1.68	2.74	4.44	6.37	7.50	13.64	12.22
24	1.87	1.66	2.73	4.46	6.37	7.59	13.92	12.20
25	1.78	1.67	2.75	4.46	6.37	7.64	13.75	12.20
26	1.26	1.66	2.75	4.44	6.37	7.86	13.61	12.20
27	1.67	1.66	2.74	4.46	6.37	7.80	13.52	12.20
28	1.50	1.66	2.73	4.45	6.37	7.81	13.62	12.21
29	1.57	1.66	2.77	4.46	6.37	7.91	14.01	12.23
30	1.39	1.67	2.75	4.45	6.37	7.87	13.53	12.20
31	1.44	1.67	2.74	4.45	6.37	7.78	13.97	12.22
32	1.36	1.67	2.73	4.45	6.37	7.46	14.01	12.23
33	1.66	1.67	2.73	4.46	6.37	7.53	13.39	12.21
34	1.51	1.66	2.77	4.46	6.37	7.63	13.99	12.22
35	1.66	1.67	2.76	4.46	6.38	7.84	13.61	12.21
36	1.48	1.67	2.73	4.46	6.38	7.63	13.99	12.21
37	1.31	1.67	2.76	4.44	6.38	7.49	13.93	12.22
38	1.55	1.66	2.74	4.45	6.37	7.50	14.12	12.22
39	1.83	1.66	2.73	4.45	6.37	7.51	13.80	12.22
40	1.48	1.65	2.75	4.45	6.37	7.85	13.38	12.22
41	1.90	1.67	2.75	4.45	6.37	7.52	13.53	12.21
42	1.78	1.66	2.74	4.46	6.37	7.88	13.35	12.21
43	1.62	1.67	2.74	4.46	6.37	7.89	14.08	12.21
44	1.39	1.66	2.75	4.45	6.37	7.78	13.42	12.23
45	1.31	1.66	2.74	4.45	6.37	7.46	13.96	12.22
46	1.92	1.67	2.73	4.45	6.37	7.54	13.26	12.20
47	1.74	1.67	2.73	4.45	6.37	7.56	13.40	12.20
48	1.88	1.66	2.75	4.46	6.37	7.75	13.71	12.22
49	1.23	1.66	2.74	4.46	6.37	7.52	13.71	12.21
50	1.95	1.66	2.76	4.45	6.37	7.59	13.62	12.20

**Table A3:** Summary of cyanoacrylate adhesive thicknesses for the tested samples.

Sample	Thickness of the Adhesive (µm)								
	Measurement Point Number								
	1	2	3	4	5	6	7	8	9
1	42.4	30.7	37.6	21.7	34.5	29.8	24.1	36.6	43.8
2	35.7	44.9	39.7	40.5	33.0	39.2	40.4	44.8	26.3
3	44.0	46.6	42.6	30.5	32.3	33.6	25.1	33.8	24.4
4	27.6	28.1	23.9	44.1	47.5	37.9	33.1	25.8	48.2
5	26.4	28.5	40.2	21.1	47.3	23.3	38.4	39.2	39.8
6	45.8	43.4	45.9	44.8	30.9	32.6	43.2	45.5	42.2
7	32.1	25.1	32.2	41.2	19.8	24.2	38.1	31.3	28.1
8	36.8	28.0	40.1	25.3	28.1	24.6	25.9	39.8	41.2
9	35.0	31.4	29.3	32.5	48.0	48.3	40.5	22.4	30.0
10	42.4	23.1	48.0	24.6	29.6	37.7	37.4	25.8	34.5
11	43.4	26.3	24.0	22.1	29.0	26.4	21.0	30.5	29.3
12	45.0	26.9	25.6	24.5	23.2	22.2	29.9	20.4	40.1
13	23.5	31.8	36.0	23.4	28.3	30.7	23.4	33.0	21.9
14	38.9	25.8	34.2	22.9	19.6	20.7	41.4	21.3	23.2
15	26.0	18.7	34.4	45.2	22.9	45.0	33.0	24.1	37.0
16	31.2	20.7	34.6	46.9	41.4	37.2	27.7	28.0	24.3
17	32.5	19.6	20.8	30.4	19.8	38.7	37.6	32.5	25.7
18	23.0	35.1	22.9	40.3	44.1	47.4	43.8	33.8	25.3
19	30.8	38.5	45.1	24.8	43.6	32.3	34.5	47.8	41.7
20	31.1	40.5	34.4	28.7	23.3	29.4	41.2	45.0	26.4

**Table A4:** Summary of structural adhesive thicknesses for tested samples.

Sample	Thickness of the Adhesive (µm)								
	Measurement Point Number								
	1	2	3	4	5	6	7	8	9
1	7.7	8.0	5.6	7.7	5.8	7.9	4.3	7.9	5.5
2	4.4	7.9	7.1	8.4	5.7	5.1	7.0	4.8	3.9
3	4.5	4.9	3.5	4.3	8.0	5.6	7.1	8.0	4.3
4	4.3	6.5	4.8	5.2	7.7	8.0	7.5	6.1	6.0
5	8.1	8.1	6.2	6.5	7.0	5.0	9.0	4.2	6.9
6	7.8	6.8	8.6	8.4	5.6	7.4	6.6	5.8	4.8
7	8.5	5.2	6.8	5.4	8.8	6.2	5.0	8.8	6.8
8	7.7	6.8	7.2	8.4	8.3	5.2	5.4	6.5	7.1
9	7.2	6.3	7.2	7.4	3.5	6.8	4.1	8.9	3.6
10	4.0	7.0	7.3	5.1	4.1	5.0	6.7	8.0	4.4
11	7.9	7.4	4.6	5.6	6.8	7.3	5.8	7.0	8.9
12	7.3	5.8	5.1	6.0	6.2	6.2	5.2	7.8	5.7
13	`	8.9	4.1	8.6	4.5	8.4	5.5	4.8	6.3
14	6.3	3.7	5.3	7.2	4.4	5.5	8.4	5.6	6.2
15	8.1	6.7	4.8	7.1	4.2	7.4	5.5	4.0	7.1
16	7.0	6.8	7.1	7.1	5.1	8.0	4.4	8.6	4.2
17	7.6	5.3	8.9	8.1	5.5	5.7	3.7	3.7	6.7
18	4.5	6.9	4.4	4.5	8.9	6.8	6.6	7.0	8.4
19	6.9	5.9	7.0	7.3	3.6	5.5	7.6	7.7	6.1
20	8.1	5.1	8.7	3.9	6.4	4.6	3.7	8.5	6.6
